# Macular Edema Without Vascular Leakage – The Diagnostic Challenge of SNIFR and its Differential Diagnoses

**DOI:** 10.1055/a-2794-2579

**Published:** 2026-03-17

**Authors:** Sophia Näther, Sandrine Zweifel, Felix Gabathuler, Frank Blaser, Peter Maloca, Katrin Fasler

**Affiliations:** 1 Department of Ophthalmology, University Hospital of Zurich, Zurich, Switzerland; 2 Department of Biomedical Engineering, University of Basel, 4123 Allschwil, Switzerland; 3 Department of Ophthalmology, University Hospital Basel, 4056 Basel, Switzerland; 4 Moorfields Eye Hospital NHS Foundation Trust, London, EC1V 2PD, United Kingdom

## Introduction

Stellate Nonhereditary Idiopathic Foveomacular Retinoschisis (SNIFR) is a rare foveomacular schisis characterized by a spoke-like separation of the outer retinal layers without vitreomacular traction or hereditary retinal disease 1 , 2 . Ober et al. first introduced SNIFR in 2014 in a case series of 17 patients, describing a predominantly unilateral, nonprogressive condition in mostly otherwise healthy middle-aged women. Spectral-domain optical coherence tomography (SD-OCT) typically shows splitting within the outer plexiform and nuclear layers with preserved inner retina. Fluorescein angiography (FA) does not show any vascular leakage. Patients are mostly asymptomatic or report mild vision reduction or metamorphopsia 1 .

Pathophysiology remains unclear. Hypotheses include a pathologically adherent posterior hyaloid, Müller cell dysfunction, or Henle fiber abnormalities. Bloch et al. reported an association with peripheral retinoschisis in eyes with persistent posterior hyaloid, suggesting subtle vitreoretinal traction may contribute to schisis formation 3 .

SNIFR is a diagnosis of exclusion, as it can mimic exudative retinal diseases such as neuroretinitis or retinal changes associated with malignant hypertension 2 , 4 , 5 . In the case of neuroretinitis, FA typically shows leakage 6 which is not observed in SNIFR. Another important differential diagnosis is congenital juvenile X-linked retinoschisis (CXLR), a genetic condition caused by mutations in the RS1 gene, almost exclusively affecting males. Unlike SNIFR, CXLR typically presents bilaterally during the first decades of life. The primary distinguishing feature is the age of onset and the bilateral nature of CXLR compared to the more variable onset and unilateral involvement seen in SNIFR. Furthermore, electroretinography (ERG) can help differentiate CXLR from SNIFR; in CXLR, ERG shows decreased b-wave amplitudes with preserved a-wave, leading to a negative pattern, whereas SNIFR generally does not show such marked ERG abnormalities 7 . Another important hereditary differential diagnosis is Enhanced S-cone syndrome, a retinal dystrophy associated with mutations in the NR2E3 gene, which leads to functional abnormalities of the S-cones. Key features distinguishing Enhanced S-cone syndrome from SNIFR include the presence of night blindness, an optically empty vitreous body, and characteristic ERG abnormalities 8 .

Various structural conditions should also be considered as potential causes of foveomacular retinoschisis. For instance, myopic degeneration can lead to foveomacular retinoschisis, which is typically tractional in nature and often develops in areas with posterior staphyloma 9 . Furthermore, foveomacular schisis has been observed in over 50% of patients with optic disc pit 10 . Additionally, certain medications have been implicated in causing central macular edema without leakage on FA. For example, niacin and anticancer agents such as docetaxel and palitaxel have been reported to induce this condition 11 , 12 . Case reports have also described macular edema triggered by other agents, including fingolimod, tamoxifen, and interferons 13 .

Multimodal imaging and functional testing are essential for excluding differential diagnosis 2 .

Here, we present two cases of macular schisis consistent with SNIFR or a SNIFR-like morphology, illustrating the diagnostic challenges and the importance of multimodal imaging for accurate classification.

## History and Signs

### Case 1

**Fig. 1:**
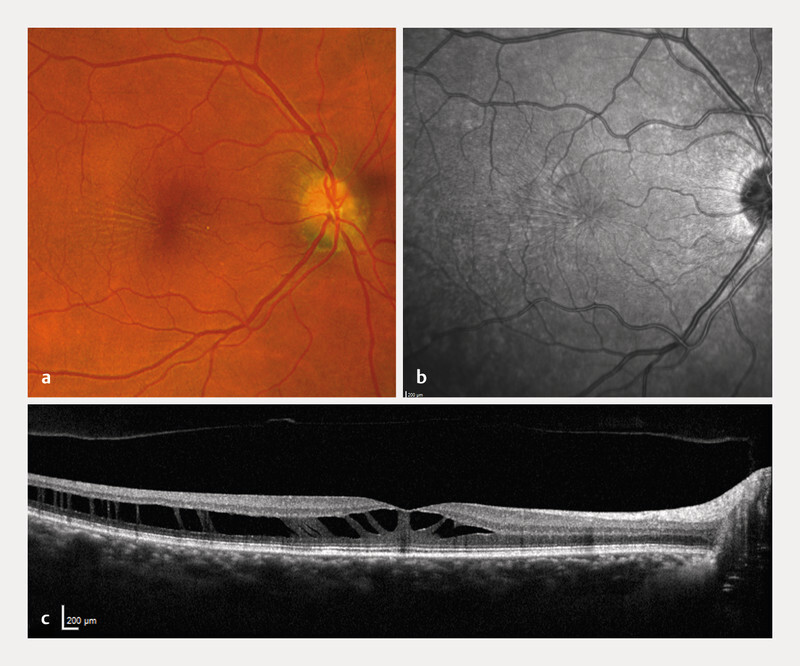
Images from the right eye of patient 1 (59-year-old woman with SNIFR). **a** Fundus false color image (Optos). **b** Infrared image, both showing radial spoking around the fovea. **c** OCT with retinoschisis (OCT – optic coherence tomography).

**Fig. 2:**
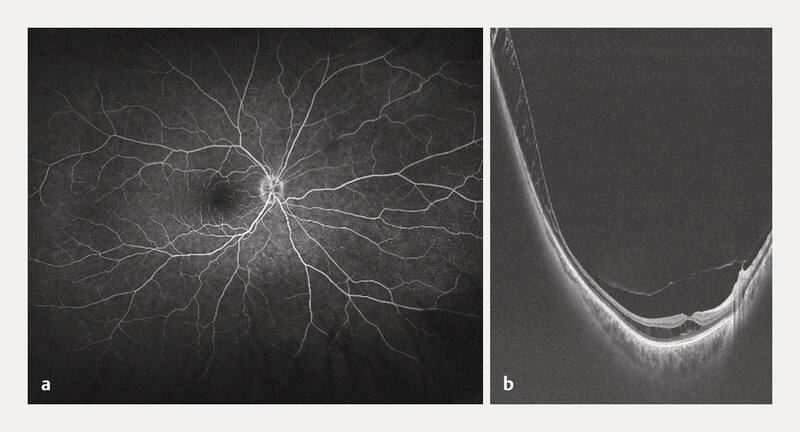
Images from the right eye of patient 1 (59-year-old woman with SNIFR). **a** FA image in the late phase (5 min 01) without leakage. **b** Widefield 20 × 24 mm OCT of patient 1, showing retinoschisis involving the macula and extending to the periphery. OCT – optical coherence tomography, FA – Fluorescein angiography.

A 59-year-old woman presented with progressive right-eye visual decline over six months (20/40 Snellen/0.5 decimal, autorefraction − 1.0 D). She had no systemic or ocular comorbidities. Fundoscopy showed subtle macular gliosis ( Fig. 1 a ); optical coherence tomography (OCT – Heidelberg Spectralis) revealed a foveal schisis extending into inner and outer retinal layers without traction ( Fig. 1 c ). FA was normal without vascular leakage ( Fig. 2 a ). Widefield OCT (Optos California) revealed peripheral extension of the schisis beyond the posterior pole ( Fig. 2 b ). No optic pit was visible. Due to the unilateral manifestation, female sex, and age, X-linked retinoschisis was considered unlikely. The diagnosis of SNIFR was made. A therapeutic trial with topical dorzolamide was initiated, treatment was discontinued after 6 weeks at the patientʼs request due to inefficacy.

### Case 2

**Fig. 3:**
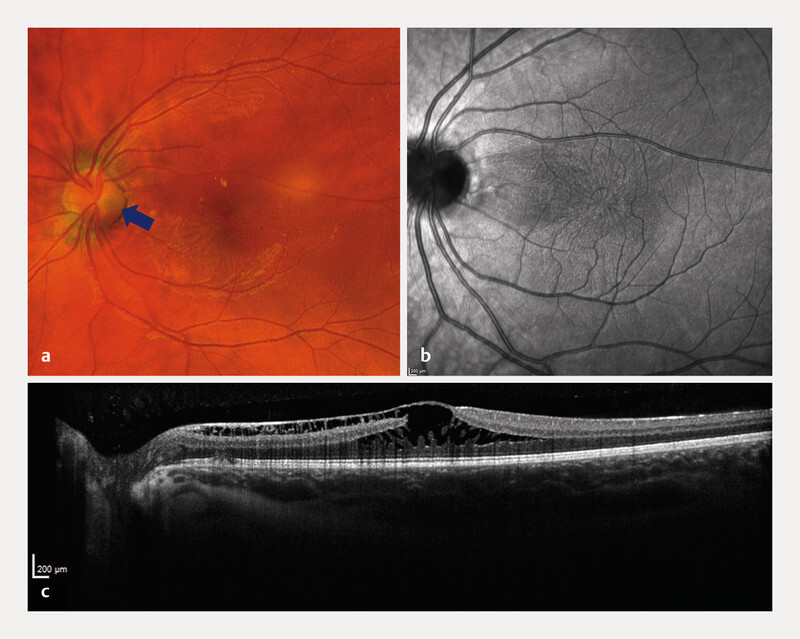
Images from the left eye of patient 2 (18-year-old woman with optic disc maculopathy). **a** Fundus false color image (Optos), optic disc with pit (blue arrow). **b** Infrared image, both showing radial spoking around the fovea. **c** OCT with retinoschisis (OCT – optic coherence tomography).

**Fig. 4:**
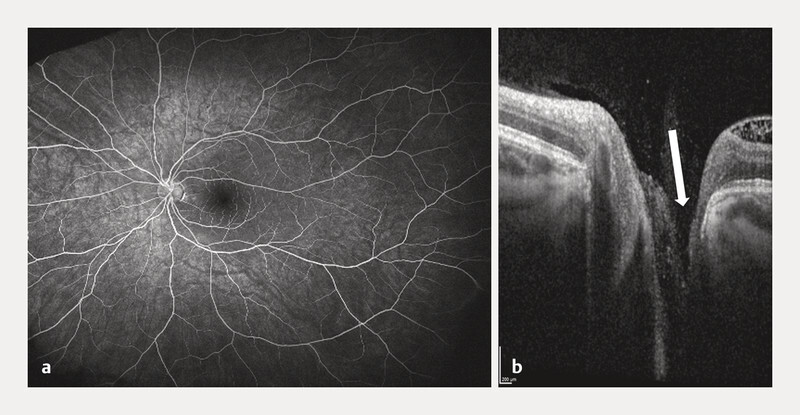
Images from the left eye of patient 2 (18-year-old woman with optic disc maculopathy). **a** FA image in the late phase (5 min 13) without leakage. **b** High resolution OCT showing optic disc pit. OCT – optic coherence tomography, FA – Fluorescein angiography.

A healthy 18-year-old woman was referred for mild unilateral visual impairment in the left eye (20/25 Snellen/0.8 decimal, autorefraction − 0.5 D), initially suspected as epiretinal membrane or pseudohole. She reported slight asymmetry in visual quality over the past one to two years. Anterior segments were unremarkable, fundoscopy showed spoke-wheel like appearance perifoveally ( Fig. 3 a ). OCT showed foveal retinoschisis in outer nuclear and plexiform layers without vitreoretinal traction ( Fig. 3 c ). FA showed no leakage ( Fig. 4 a ). Full-field ERG (Diagnosys LLC) was normal, multifocal ERG revealed a borderline response in ring 1 of the left eye. Upon closer inspection, a subtle excavation of the optic disc was detected; high-resolution OCT over the disc confirmed an optic disc pit ( Fig. 4 b ). Widefield OCT showed no peripheral schisis. The final diagnosis was optic pit maculopathy rather than SNIFR.

## Therapy and Outcome

Management of macular schisis without vascular leakage is largely conservative. Case 1 showed structural stability with local dorzolamide over 6 weeks, no visual improvement, and remained stable after discontinuation. In Case 2, the diagnosis of optic pit maculopathy precluded further intervention; the schisis remained stable without therapy.

## Discussion

Our two cases illustrate the heterogeneity and diagnostic complexity of foveomacular retinoschisis. Case 1 represents a classic SNIFR phenotype: unilateral foveal and perifoveal schisis in a middle-aged woman, involving also the peripheral retina, absence of leakage on FA, and stability over time. The limited response to topical dorzolamide in our patient corresponds with the report of Schildroth et al. and stands in contrast to the case described by Ajlan et al. 14 , 15 . The mechanism remains speculative; inhibition of carbonic anhydrase in Müller cells may enhance subretinal fluid resorption, but most cases stabilize spontaneously, as also already noted by Ober et al. in 2014 1 .

Therapeutic experience is limited. Ajlan et al. reported structural improvement with topical dorzolamide (4), whereas other series showed no change 14 . Most authors recommend observation due to the generally benign nature of SNIFR.

Case 2 highlights an important differential diagnosis: optic disc maculopathy can closely mimic the OCT appearance of SNIFR, especially when the optic pit is not readily visible on fundoscopy and schisis cavities are confined to the outer layers. As Light et. al pointed out, high-resolution imaging of the optic nerve is essential to exclude congenital disc anomalies before diagnosing idiopathic forms 2 . Typically, an optic disc pit presents as a unilateral, round or oval, greyish excavation located near the margin of the optic disc. The pathophysiology of optic disc pit maculopathy remains poorly understood, and the origin of the associated fluid is still unclear. Both vitreous fluid and cerebrospinal fluid have been proposed as potential sources 16 . Optic disc pit maculopathy typically occurs in the third or fourth decade of life, the symptoms are usually non-specific 17 .

Recent findings by Feo et al. provide further insights into the pathogenesis of SNIFR. Their longitudinal imaging demonstrated that mid-peripheral schisis can progress centripetally toward the fovea, sometimes forming SNIFR-like lesions, and that subtle vitreoretinal traction in the mid-periphery may serve as the initiating factor 18 . In our series, widefield OCT identified peripheral extension in one patient, supporting the concept of a tractional continuum between peripheral and central schisis.

While most patients with SNIFR retain good visual acuity, as in our Case 1, long-term monitoring remains advisable. Feo et al. also described CARPET variant with central neurosensory detachment and lamellar defects, which may progress more aggressively and occasionally require vitrectomy 18 . None of our cases showed such features but awareness of this potential evolution is important for clinical decision-making.

Overall, the absence of exudation, the characteristic OCT architecture, unilateral manifestation and the stable course distinguish SNIFR from cystoid macular edema and hereditary retinoschisis. The combination of SD-OCT, widefield OCT, and functional testing such as ERG is crucial for accurate classification. From a therapeutic point of view, conservative management remains appropriate in most cases; topical carbonic anhydrase inhibitors may be considered on a case-by-case basis, although evidence remains anecdotal.

In summary, our two cases demonstrate different causes of macular schisis without vascular leakage. SNIFR remains an underrecognized condition with a largely benign but chronic course. Further longitudinal studies are warranted to clarify its pathophysiology and define optimal management strategies.
